# Food, nutrition and sustainability education in Australian primary schools: a cross-sectional analysis of teacher perspectives and practices

**DOI:** 10.1186/s13690-024-01449-4

**Published:** 2024-11-22

**Authors:** Jessica V Kempler, Claire Margerison, Janandani Nanayakkara, Alison Booth

**Affiliations:** Deakin University Institute for Physical Activity and Nutrition, 75 Pigdons Road Waurn Ponds, Victoria, 3216 Australia

**Keywords:** Nutrition education, Food education, Sustainability education, Food literacy, Primary school, Primary school teacher

## Abstract

**Background:**

Healthy eating patterns from sustainable food systems are crucial for population and planetary health. Primary schools are opportune settings for teaching children about food, nutrition and sustainability (FNS) though little is known about the delivery of FNS education in this sector. This study aimed to analyse current approaches to FNS education in Australian primary schools.

**Methods:**

A cross-sectional online survey with closed- and open-ended questions collected data about (i) teacher perceptions and attributes regarding FNS education; (ii) FNS teaching practices; and (iii) factors influencing FNS education. Statistical analyses were conducted using STATA including descriptive statistics and chi-square analyses to test for associations between categorical variables. Qualitative content and thematic analyses of open-ended questions were conducted using NVivo 14.

**Results:**

Participants were 413 Australian primary school teachers. Teachers considered FNS education as equally important to most curriculum subjects, though frequency of FNS education was low. Less than a third of teachers were trained in FNS education, had access to funding for FNS-related activities or were from schools with policies about including FNS education in the curriculum. There was a significant association between frequency of FNS education and teacher training, access to funding and presence of FNS curriculum policies (all *p* < 0.001). Teachers who were trained to teach nutrition, food skills or food sustainability (all *p* < 0.05) were more likely to teach this as both stand-alone and cross-curricular subjects. Teachers described personal factors (workload, stress, scope of practice) that influenced their FNS teaching practices, as well as factors related to students’ families (family responsibility for FNS education, family food practices, family engagement in FNS activities), the curriculum (overcrowding, prioritisation, access to resources) and the broader school environment (time, facilities, funding, training).

**Conclusions:**

Strengthening the position of FNS education in the primary school sector is an important next step for public health research, policy and practice. Researchers and policy makers should explore future opportunities for training, funding and policy approaches that prioritise FNS within the primary school curriculum and in everyday teaching practice.

**Supplementary Information:**

The online version contains supplementary material available at 10.1186/s13690-024-01449-4.

**Table:** 

Text box 1. Contributions to the literature
• Public health literature indicates school-based food interventions improve children’s food-related behaviours and population health outcomes.
• Very few studies have analysed actual practices in school-based food education outside of time-limited and siloed interventions.
• This study addresses an important gap in published literature by reporting on current practices in primary school food and nutrition education with a specific focus on food sustainability.
• This may support public health researchers, policy makers and practitioners to address food-related behavioural and social determinants of health through integrated strategies within the school setting.
• This may help improve population health outcomes for school children, families and broader communities.

## Background

Healthy eating patterns from sustainable food systems are essential for population and planetary health. Whilst healthy food behaviours help prevent and manage non-communicable diseases, sustainable food systems preserve environmental, social and economic resources for current and future generations [1].

Currently, global diets do not meet health-based recommendations [2–5] and result in greenhouse gas emissions, terrestrial acidification, fresh-water eutrophication and depletion of non-renewable resources [6, 7]. Developing food literacy is an important mechanism for addressing these challenges [8, 9]. Whilst several food literacy models exist [8–10], the concept can be described as the inter-related knowledge, skills and behaviours that empower people to interact with food in a way that supports personal health and environmental sustainability.

The primary school aged years (5–12 years) are a critical time for developing food literacy [11]. During this crucial period of growth and development, food habits that may persist into later years are formed [12, 13], ecological awareness is being established [14], and children develop a greater sense of agency to make autonomous food choices [15]. Strategies are needed to help children navigate these complex changes.

Systematic reviews suggest school-based food literacy interventions can positively impact children’s nutrition knowledge [16, 17], dietary behaviours [16–20] and anthropometric measures [18, 21, 22]. Consequently, international agencies including the World Health Organisation [23, 24] and Food and Agriculture Organisation [25] have advocated for schools as opportune places for building children’s food literacy.

In several countries including Australia [26, 27], government funding and compulsory attendance means primary schools have intensive contact with most children during their formative years, irrespective of ethnicity, culture or socioeconomic position [21]. They offer multiple formal and informal opportunities for food-related learning [28–31], and employ degree-qualified teachers who are key agents for promoting children’s food literacy.

Whilst there has been an emergence of food, nutrition and sustainability topics within national [32–34] and state [35] jurisdiction curricula, little is known about how this translates into classroom teaching. Only a limited number of small studies have investigated teachers’ nutrition education practices in primary schools [36–41], and these have not considered food sustainability. Whilst few studies have explored teacher perceptions, attributes and other factors that impact the delivery of food and nutrition education [36, 42, 43], little is known about what influences delivery of food sustainability education. Collectively, the gaps in evidence regarding current practices in primary school food, nutrition and sustainability education indicate this field of research is in its formative stage. To our knowledge, this is among the first study of its kind to address these gaps.

## Aim and objectives

The primary aim of this study was to analyse current approaches to food, nutrition and sustainability (FNS) education in Australian primary schools. The objectives were to analyse (i) teacher perceptions and attributes regarding FNS education; (ii) FNS teaching practices; and (iii) factors influencing FNS education in Australian primary schools. The secondary aim was to determine whether these were associated with school characteristics including school type, size, socioeconomic position (SEP) and rurality.

## Methods

### Study design and setting

A cross-sectional mixed methods survey of Australian primary school teachers was undertaken to analyze approaches to FNS education. The study is part of a larger transnational analysis of FNS teaching practices in Australia, Sweden and the United Kingdom. Here we report on a sub-set of data collected from Australian respondents. Methods and results are reported according to the Strengthening the Reporting of Observational Studies in Epidemiology (STROBE) Statement [44].

### Participants

Eligible participants were current classroom teachers of primary school students in Foundation (Prep) – Grade Six (approximately 5–12 years old) at a registered Australian school. Respondents were excluded if they were not a classroom teacher (for example, other school staff) or did not work at a registered school (for example, homeschooling).

### Sample size

Given this study is part of a transnational analysis, an adequate sample size was necessary to detect the magnitude of associations between variables across countries. The STATA sample size calculator [45] determined a sample of 388 respondents per country would detect a 10% difference in country responses with 80% power, as consistent with contemporary research [46]. This would also allow estimation of prevalences (for example, perceptions about the importance of FNS education) with a maximum 95% confidence interval (CI) width of ± 5%. The target sample size for this study was therefore 388 participants.

### Recruitment and consent

Australian respondents were recruited via convenience sampling between August 2022-October 2023. Advertisements with an online survey link were circulated via (i) social media campaigns; (ii) relevant organisational mailing lists; (iii) education department school listings; and (iv) alumni listings from Deakin University education courses. Organisational consent was obtained from all agencies prior to advertisement.

Teachers who received the advertisement and clicked on the embedded link were directed to a voluntary, self-administered survey. On selecting *‘Australia’* as their country, respondents were re-directed to an Australia-specific survey which included embedded consent. Respondents who consented to participate, and who responded ‘yes’ to the screening question ‘*Are you currently a classroom teacher of primary level students (Foundation - Grade 6) at a school in Australia?’* could complete the survey.

### Survey instrument

#### Transnational survey

This survey was designed to explore FNS education practices in Australian primary schools and compare practices across countries. Due to the novelty of the study, a transnational survey tool was purpose-designed by the research team, based on published tools that have assessed school food sustainability [47] and nutrition [48, 49] practices and environments. Questions were adapted and added to meet specific research objectives. As there is no formal definition of what comprises FNS education, we utilised the following broad definitions:

- Nutrition education: teaching students about nutritious foods and eating patterns that promote good health and reduce the risk of disease.
- Food skills education: teaching students to plan, purchase, store, prepare and cook nutritious food.
- Food sustainability education: teaching students to use nutritious food in a way that does not waste natural resources and can be continued into the future without harming our environment or health.

The survey was tested for face validity by six researchers with expertise in school-based FNS education, including two teachers. Minor amendments to improve clarity were incorporated into the final questions. The survey was built into the secure REDCap (Research Electronic Data Capture) platform [50], and was designed to be completed in approximately 15 min.

#### Current study survey measures

This study reports on Australian participant responses to a subset of questions from the transnational survey which captured data about (i) teacher perceptions and attributes regarding FNS education; (ii) FNS teaching practices; (iii) factors influencing FNS education; and (iv) school and teacher demographics (*n* = 10 questions). Descriptive quantitative questions were presented using matrix format with 3-point (*n* = 1) and 5-point (*n* = 8) Likert scales and both single-select (*n* = 8) and multi-select (*n* = 5) responses (question topics are summarised in Table 1, see supplementary file for exact wording). For some questions, prompts (*‘why…’*,* ‘how…’* or *‘please specify…’*) were used to glean specific information. Two open-ended questions sought additional information deemed important by respondents (*‘Is there anything else you think is important for us to know about…?*’). We anticipated that insights from spontaneous responses to these questions would reflect important perspectives from teachers that could not be captured using predefined, researcher-generated questions and response scales [51].

**Table 1:** Summary of question domains, topics and response types

Domain	Question topics	Response types
Teacher perceptions and attributes regarding FNS education	•Teacher and school responsibilities •Importance of FNS education •Understanding, knowledge/skills, confidence, enjoyment and training to teach FNS	5-point and 3-point Likert scales, single-select
FNS teaching practices	•Frequency of FNS education •Person who teaches FNS •Teaching approaches (cross-curriculum vs. stand-alone subject) •FNS educational resources, materials and information sources	Single-select / multi-select
Factors influencing FNS education	•Family engagement •Barriers •Funding •Policies	Single-select / multi-select
School and teacher demographics	School characteristics •State, postcode, school type, number of enrolments Teacher characteristics: •Grades taught, years of teaching experience, years in current role, postcode, age, gender identity	Single-select / multi-select

### Data analysis

#### Statistical analysis

Statistical analyses were conducted using STATA. Descriptive statistics (frequencies and proportions) were generated for all categorical variables. Sample characteristics were presented as frequencies and percentages, whilst teacher perceptions and attributes, FNS teaching practices and factors influencing FNS education were presented as percentages with 95%CI. For questions about FNS teaching materials and information sources (asked only to teachers who taught FNS), frequencies and percentages were presented.

Area-level SEP was determined using the Index of Relative Socioeconomic Advantage and Disadvantage (IRSAD) [52]. Decile scores were allocated based on postcode (1 = greatest disadvantage; 10 = greatest advantage) and categorised as low-SEP (IRSAD 1–3), medium-SEP (IRSAD 4–6) or high-SEP (IRSAD 7–10). School type was dichotomised as government or non-government (including independent, Catholic and other schools). School rurality was determined using postcode matching to the Australian Statistical Geography Standard [53] which classifies geographic areas as a major city, inner/outer regional Australia or remote/very remote Australia. Rurality was then dichotomised as major city or regional city. School size was grouped into tertiles. Responses to matrix questions with 5-point Likert scales were grouped into three categories (agree/neither agree nor disagree/disagree). Frequency of FNS education was grouped into 3 categories (frequently/infrequently/never). Where data were missing, responses to qualitative prompts were considered and coded if possible. Where this was not possible, data were coded as ‘missing’.

Chi-square tests were used to analyse associations between FNS practices (frequency, teaching approaches, teacher training, presence of FNS-related policies and access to funding) and school demographics (school type, size, SEP and rurality). Statistical significance was set a priori at *p* < 0.05. Post hoc analyses using contingency tables and adjusted standardised residuals were used to determine combinations of categories contributing the most to significant associations.

#### Qualitative analysis

Qualitative analyses were conducted using NVivo 14 [54]. Content analysis was used to analyse text responses to prompt questions. Codes were defined in NVivo and text was coded and counted.

Braune and Clarke’s approach to inductive thematic analysis was used to explore open-ended questions [55]. An open coding technique was used to assign codes to raw data extracts. Codes were not pre-defined but were developed and labelled using an inductive process. To minimize bias, a 10% sample of survey response files (*n* = 41) was independently coded by the lead author and one researcher outside of the research team and consensus on grouping codes was reached. This verification process has been previously used in inductive thematic analysis of qualitative survey research in food education [56]. Data extracted from the remaining survey responses were coded by the lead author. Codes were systematically categorized to determine common themes and their trends and patterns. Through iterative analysis, themes were refined and named, and then rechecked to ensure they accurately reflected raw data extracts.

## Results

### Respondents

**Fig. 1:**
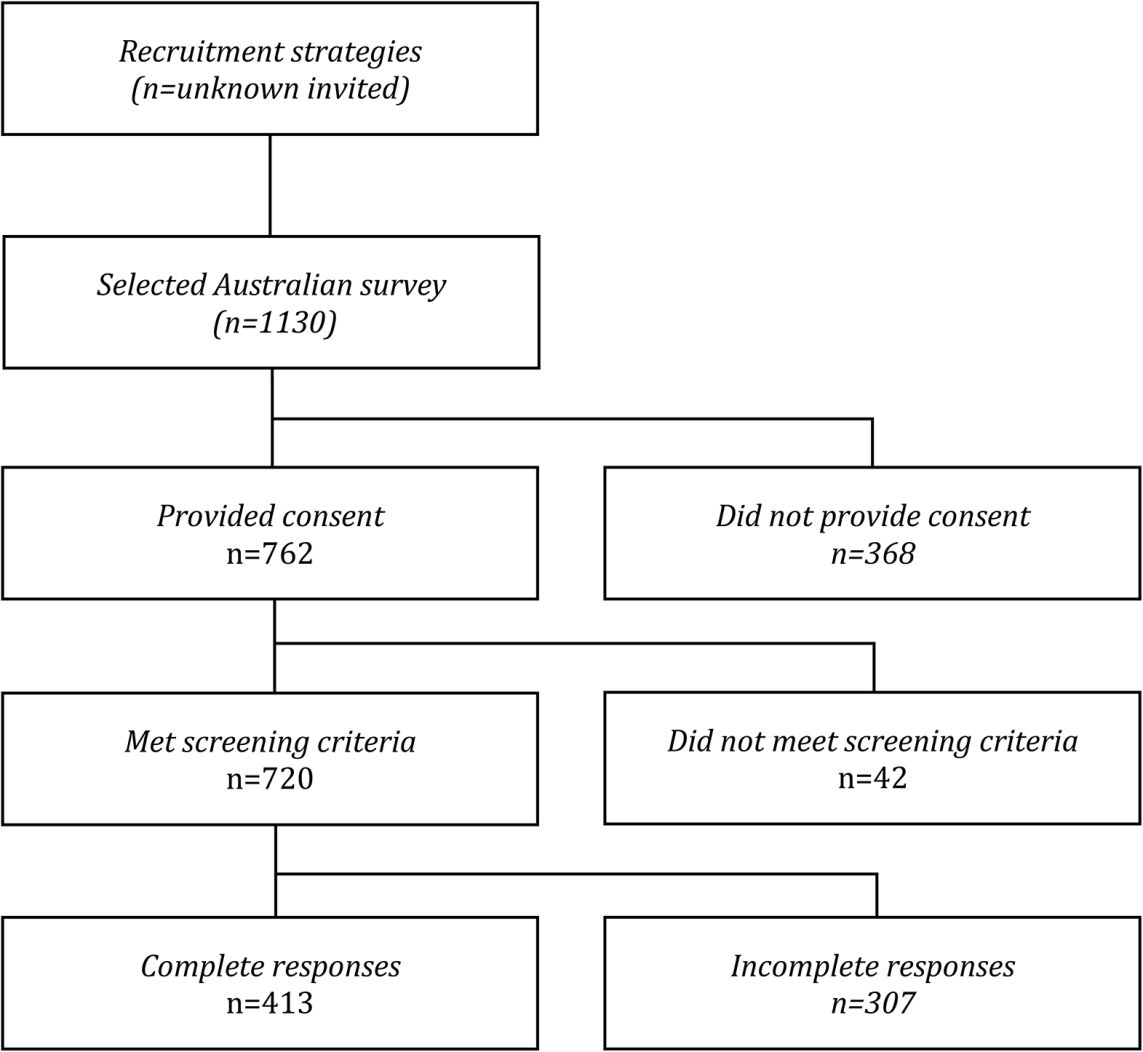
Participant recruitment and survey responses

A total of 413 primary school teachers completed the survey and were included in the analysis (Fig. 1).

Respondents were mostly female (*n* = 357, 86%), working in Victoria (*n* = 238, 57%) and teaching at a government school (*n* = 282, 68%). School and teacher characteristics are outlined in Table 2.

**Table 2:** School and teacher characteristics (*n* = 413)

	Proportion *n* (%)
**School characteristics**	
**State / Territory**	
Victoria	237 (57.4)
Queensland	63 (15.3)
New South Wales	45 (10.9)
South Australia	22 (5.3)
Western Australia	14 (3.4)
Australian Capital Territory	13 (3.1)
Northern Territory	6 (1.5)
Tasmania	5 (1.2)
Missing	8 (1.9)
**SEP**	
Low (IRSAD decile 1–3)	82 (19.9)
Medium (IRSAD decile 4–6)	102 (24.7)
High (IRSAD decile 7–10)	219 (53.0)
Missing	10 (2.4)
**Rurality**	
Major City of Australia	262 (63.4)
Inner Regional Australia	81 (19.6)
Outer Regional Australia	44 (10.7)
Remote Australia	9 (2.2)
Very Remote Australia	6 (1.5)
Missing	11 (2.7)
**School type**	
Government/public	282 (68.3)
Independent/private	51 (12.3)
Catholic	77 (18.6)
Other	3 (0.7)
**Student enrolments**	
< 24	14 (3.4)
25–49	11 (2.7)
50–99	23 (5.6)
100–199	39 (9.4)
200–299	61 (14.8)
300–399	65 (15.7)
400–499	51 (12.3)
500–599	58 (14.0)
600–699	38 (9.2)
700–799	14 (3.4)
800–899	14 (3.4)
900–999	13 (3.1)
> 1000	12 (2.9)
**Teacher characteristics**	
**Grades taught#**	
Foundation	122 (29.5)
Grade 1	130 (31.5)
Grade 2	131 (31.7)
Grade 3	143 (34.6)
Grade 4	164 (39.7)
Grade 5	151 (36.6)
Grade 6	141 (34.1)
**Time as a teacher (years)**	
< 1	16 (3.9)
1–5	86 (20.8)
6–10	98 (23.7)
11–15	69 (16.7)
16–20	44 (10.7)
21–25	37 (9.0)
> 25	63 (15.3)
**Time in current role (years)**	
< 1	84 (20.3)
1–5	175 (42.4)
6–10	87 (21.1)
11–15	31 (7.5)
16–20	14 (3.4)
21–25	11 (2.7)
> 25	11 (2.7)
**Age (years)**	
18–24	20 (4.8)
25–34	112 (27.1)
35–44	112 (27.1)
45–54	92 (22.3)
55–64	73 (17.7)
65+	4 (1.0)
**Gender**	
Woman	356 (86.2)
Man	42 (10.2)
Gender diverse	1 (0.2)
Non-binary	2 (0.5)
Prefer to self-describe	2 (0.5)
Prefer not to specify	10 (2.4)
**SEP**	
Low (IRSAD 1–3)	54 (13.1)
Medium (IRSAD 4–6)	112 (27.1)
High (IRSAD 7–10)	234 (56.7)
Missing	13 (3.1)

For all characteristics percentages may not sum to 100% due to rounding

# For ‘grades taught’ percentages do not sum to 100% due to participants teaching in more than one grade level

IRSAD = Index of Relative Socio-Economic Advantage and Disadvantage

SEP = Socioeconomic Position

### Quantitative results

#### Teacher perceptions and attributes regarding FNS education

##### Teacher and school responsibilities

Most teachers agreed FNS should be taught in the curriculum (86.0%; 95%CI = 82.2%, 89.0%) and that schools should have policies about this (69.5%; 95%CI = 64.9%, 73.8%). They agreed teachers should role model healthy eating (80.1%; 95%CI = 76.0%, 83.7%) and encourage students to eat healthy food (88.9%; 95%CI = 85.4%, 91.6%).

##### Importance of FNS education

Most teachers reported it is extremely/very important to teach students about nutrition (83.8%; 95%CI = 79.9%, 87.0%), food skills (69.7%; 95%CI = 65.1%, 74.0%) and food sustainability (74.1%; 95%CI = 69.6%, 78.1%). Compared to other curriculum areas, most considered FNS education as important as health and physical education (HPE) (86.9%; 95%CI = 83.3%, 89.9%), mental health and wellbeing (73.8%; 95%CI = 69.4, 77.9%), humanities and social sciences (HASS) (72.2%; 95%CI = 67.6%, 76.3%), science (70.9%; 95%CI = 66.4%, 75.1%), arts (70.0%; 95%CI = 65.4%, 74.2%) and technologies (67.8% = 95%CI: 63.1%, 72.1%). Over half considered FNS less important than English (53.5%; 95%CI = 48.7%, 58.3%) and maths (53.0%; 95%CI = 48.2%, 57.8%), whilst a third (31.0%; 95%CI = 26.7%, 35.6%) reported it is more important than languages.

##### Understanding, knowledge and skills, confidence, enjoyment and training

Teachers reported high levels of understanding, confidence, knowledge and skills and enjoyment regarding FNS education. Less than a third had undertaken training to teach this (Table 3). Teachers in non-government schools were more likely to be trained to teach food sustainability compared to teachers in government schools (*p* = 0.022, see Table 4), whilst teachers from smaller schools were more likely to be trained to teach food skills (*p* = 0.010). Similarly, teachers who had access to funding for training were more likely to have completed training to teach nutrition (59.8% vs. 22.1%), food skills (50.0% vs. 16.0%) and food sustainability (47.6% vs. 18.7%) (all *p* < 0.001). Teachers from schools with FNS curriculum policies were more likely to be trained to teach both food sustainability (31.4% vs. 16.4%; *p* = 0.025) and nutrition (37.3% vs. 23.1%; *p* = 0.038) than teachers from schools without such policies in place. School SEP and rurality were not predictors for teacher training.

**Table 3:** Teacher understanding, knowledge and skills, confidence, enjoyment and training to teach FNS (*n* = 413)

	Agree / strongly agree		Neither agree nor disagree		Disagree / strongly disagree	
	*n*	% (95%CI)	*n*	% (95%CI)	*n*	% (95%CI)
**I have a good understanding of**:						
Nutrition	382	92.5 (89.5, 94.7)	18	4.4 (2.8, 6.8)	13	3.1 (1.8, 5.4)
Food skills	369	89.3 (86.0, 92.0)	33	8.0 (5.7, 11.0)	11	2.7 (1.5, 4.8)
Food sustainability	325	78.7 (74.5, 82.4)	60	14.5 (11.4, 18.3)	28	6.8 (4.7, 9.7)
**I have the knowledge and skills required to teach my students about**:						
Nutrition	312	75.5 (71.2, 79.5)	53	12.8 (9.9, 16.4)	48	11.6 (8.9, 15.1)
Food skills	291	70.5 (65.9, 74.7)	68	16.5 (13.2, 20.4)	54	13.1 (10.1, 16.7)
Food sustainability	258	62.5 (57.7, 67.0)	88	21.3 (17.6, 25.5)	67	16.2 (13.0, 20.1)
**I am confident in my ability to teach my students about**:						
Nutrition	358	86.7 (83.0, 89.6)	26	6.3 (4.3, 9.1)	29	7.0 (4.9, 9.9)
Food skills	340	82.3 (78.3, 85.7)	47	11.4 (8.7, 14.8)	26	6.3 (4.3, 9.1)
Food sustainability	305	73.8 (69.4, 77.9)	64	15.5 (12.3, 19.3)	44	10.7 (8.0, 14.0)
**I enjoy/would enjoy teaching my students about**:						
Nutrition	352	85.2 (81.5, 88.3)	34	8.2 (5.9, 11.3)	27	6.5 (4.5, 9.4)
Food skills	339	82.1 (78.1, 85.5)	41	9.9 (7.4, 13.2)	33	8.0 (5.7, 11.0)
Food sustainability	324	78.5 (74.2, 82.2)	58	14.0 (11.0, 17.8)	31	7.5 (5.3, 10.5)
**I have undertaken training and/or professional development to teach my students about**:						
Nutrition	122	29.5 (25.3, 34.1)	53	12.8 (9.9, 16.4)	238	57.6 (52.8, 62.3)
Food skills	94	22.8 (19.0, 27.1)	68	16.5 (13.2, 20.4)	251	60.8 (56.0, 65.4)
Food sustainability	101	24.5 (20.5, 28.8)	72	17.4 (14.1, 21.4)	240	58.1 (53.3, 62.8)

**Table 4:** Associations between FNS training, frequency of education, teaching approach, policies, funding and school demographics

	School size (enrolments)				School type			School SEP				School rurality		
	0-299	300–599	> 600	*p*	Government	Non-government	*p*	Low	Medium	High	*p*	Major city	Regional city	*p*
Training to teach nutrition (*n* = 413)														
Disagree	77 (52.0)	105 (60.3)	56 (61.5)	0.388	173 (61.4)	65 (49.6)	0.080	43 (52.4)	65 (63.7)	127 (58.0)	0.600	154 (58.8)	80 (57.1)	0.817
Neither	19 (12.8)	24 (13.8)	10 (11.0)		33 (11.7)	20 (15.3)		12 (14.6)	10 (9.8)	30 (13.7)		35 (13.4)	17 (12.1)	
Agree	52 (35.1)	45 (25.9)	25 (27.5)		76 (27.0)	46 (35.1)		27 (32.9)	27 (26.5)	62 (28.3)		73 (27.9)	43 (30.7)	
**Training to teach food skills** (***n* = 413)**														
Disagree	77 (52.0)	111 (63.8)	63 (69.2)	0.010	179 (63.5)	72 (55.0)	0.192	47 (57.3)	67 (65.7)	135 (61.6)	0.673	166 (63.4)	82 (58.6)	0.598
Neither	23 (15.5)	30 (17.2)	15 (16.5)		41 (14.5)	27 (20.6)		13 (15.9)	17 (16.7)	34 (15.5)		41 (15.7)	23 (16.4)	
Agree	48 (32.4)	33 (19.0)	13 (14.3)		62 (22.0)	32 (24.4)		22 (26.8)	18 (17.7)	50 (22.8)		55 (21.0)	35 (25.0)	
**Training to teach food sustainability** (***n* = 413)**														
Disagree	83 (56.1)	102 (58.6)	55 (60.4)	0.962	176 (62.4)	64 (48.9)	0.022	48 (58.5)	65 (63.7)	124 (56.6)	0.414	156 (59.5)	80 (57.1)	0.560
Neither	26 (17.6)	31 (17.8)	15 (16.5)		47 (16.7)	25 (19.1)		11 (13.4)	19 (18.6)	39 (17.8)		47 (17.9)	22 (15.7)	
Agree	39 (26.4)	41 (23.6)	21 (23.1)		59 (20.9)	42 (32.1)		23 (28.1)	18 (17.7)	56 (25.6)		59 (22.5)	38 (27.1)	
**Frequency of FNS education**														
Never	14 (9.7)	25 (14.6)	14 (15.6)	0.146	40 (14.2)	13 (10.4)	0.551	5 (6.3)	15 (15.0)	32 (14.8)	0.068	34 (13.2)	18 (13.1)	0.005
Infrequently	74 (51.0)	98 (57.3)	52 (57.8)		153 (54.5)	71 (56.8)		43 (53.8)	51 (51.0)	128 (59.3)		158 (71.5)	63 (46.0)	
Frequently	57 (39.3)	48 (28.1)	24 (26.7)		88 (31.3)	41 (32.8)		32 (40.0)	34 (34.0)	56 (25.9)		66 (25.6)	56 (40.9)	
**Teaching approach**														
Stand-alone only	26 (20.8)	27 (20.3)	17 (23.9)	0.118	46 (20.9)	24 (22.0)	0.907	11 (15.5)	21 (27.3)	33 (19.2)	0.014	42 (20.4)	23 (20.4)	0.058
Both stand-alone and cross-curriculum	62 (49.6)	49 (36.8)	34 (47.9)		96 (43.6)	49 (45.0)		39 (54.9)	37 (48.1)	65 (37.8)		82 (39.8)	59 (52.2)	
Cross-curriculum only	37 (29.6)	57 (42.9)	20 (28.2)		78 (35.5)	36 (33.0)		21 (29.6)	19 (24.7)	74 (43.0)		82 (39.8)	31 (27.4)	
**Presence of FNS policies**														
Yes	36 (24.3)	55 (31.6)	27 (29.7)	0.113	71 (25.2)	47 (35.9)	0.047	16 (19.5)	37 (36.3)	61 (27.9)	0.046	72 (27.5)	42 (30.0)	0.116
Unsure	56 (37.8)	62 (35.6)	43 (47.3)		111 (39.4)	50 (38.2)		32 (39.0)	31 (30.4)	93 (42.5)		111 (42.4)	45 (32.1)	
No	56 (37.8)	57 (32.8)	21 (23.1)		100 (35.5)	34 (26.0)		34 (41.5)	34 (33.3)	65 (29.7)		79 (30.2)	53 (37.9)	
**Funding for FNS activities**														
Yes	50 (33.8)	49 (28.2)	24 (26.4)	0.395	72 (25.5)	51 (38.9)	0.008	28 (34.2)	27 (26.5)	61 (27.9)	0.470	77 (29.4)	39 (27.9)	0.747
No	98 (66.2)	125 (71.8)	67 (73.6)		210 (74.5)	80 (61.1)		54 (65.9)	75 (73.5)	158 (72.2)		185 (70.6)	101 (72.1)	
**Funding for training to develop own FNS knowledge**														
Yes	31 (21.0)	26 (14.9)	21 (23.1)	0.200	42 (14.9)	36 (27.5)	0.002	14 (17.1)	23 (22.6)	36 (16.4)	0.401	44 (16.8)	29 (20.7)	0.331
No	117 (79.0)	148 (85.1)	70 (76.9)		240 (85.1)	95 (72.5)		68 (82.9)	79 (77.5)	183 (83.6)		218 (83.2)	111 (79.3)	
**Funding for training to teach FNS**														
Yes	32 (21.6)	28 (16.1)	22 (24.2)	0.234	48 (17.0)	34 (26.0)	0.034	15 (18.3)	21 (20.6)	41 (18.7)	0.904	48 (18.3)	29 (20.7)	0.561
No	116 (78.4)	146 (83.9)	69 (75.8)		234 (83.0)	97 (74.0)		67 (81.7)	81 (79.4)	178 (81.3)		214 (81.7)	111 (79.3)	

#### FNS teaching practices

##### Frequency, teaching role and teaching approach

FNS was most commonly taught once or twice a year (27.6%; 95%CI: 23.5%, 32.1%) or once or twice a term (26.2%; 95%CI: 22.1, 30.6%) (Table 5). FNS was taught more frequently by teachers in regional area schools vs. major city schools (*p* = 0.005). FNS was also taught more frequently when teachers had undertaken training in nutrition (50.4% vs. 19.3%), food skills (49.5% vs. 20.7%) or food sustainability (52.0% vs. 21.3%) (all *p* < 0.001), had access to funding for training (48.8% vs. 27.6%; *p* = 0.001) or FNS class activities (49.6% vs. 24.2%; *p* < 0.001) or were from schools with FNS curriculum policies (45.3% vs. 23.7%; *p* < 0.001). Frequency of FNS education was not associated with school type, size or SEP. Most teachers (74.1%; 95%CI = 69.6%, 78.1%) reported insufficient time is spent delivering FNS education.

**Table 5:** Frequency, teaching responsibilities and teaching approaches regarding FNS education

	*n*	% (95%CI)
**Frequency of FNS education** (*n* = 413*)		
A few times a week	29	7.0 (4.9, 9.9)
Once a week	57	13.8 (10.8, 17.5)
Once a fortnight	38	9.2 (6.8, 12.4)
Once or twice a term	108	26.2 (22.1, 30.6)
Once or twice a year	114	27.6 (23.5, 32.1)
Never but I am interested in this being taught	43	10.4 (7.8, 13.8)
Never and I am not interested in this being taught	9	2.2 (1.1, 4.1)
Other	15	3.6 (2.2, 5.9)
**Person who teaches FNS (** ***n*** = 361*)		
Only me	146	40.4 (35.5, 45.6)
Me and others	183	50.7 (45.5, 55.8)
Only others	32	8.9 (6.3, 12.3)
**Teaching approach (** ***n*** = 329*)		
Stand-alone subjects only	70	21.3 (17.2, 26.1)
Both stand-alone and cross-curriculum subjects	145	44.1 (38.8, 49.5)
Cross-curriculum subjects only	114	34.7 (29.7, 40.0)

*Percentages may not sum to 100% due to rounding

Three hundred and twenty nine teachers taught FNS themselves either solely (40.4%; 95%CI = 35.5%, 45.6%), or in addition to others (50.7%; 95%CI = 45.5%, 55.8%), for example a specialist teacher. Of these, 44.1% (95%CI = 38.8, 49.5) taught FNS as both a stand-alone and cross-curriculum subject. Teachers from schools in low SEP areas were more likely to teach FNS using both approaches than teachers in high SEP areas (*p* = 0.014), though there was no association between teaching approach and school type, size or rurality. Teachers who were trained to teach nutrition (51.8% vs. 37.2%; *p* = 0.006), food skills (58.3% vs. 37.3%; *p* = 0.004) or food sustainability (58.2% vs. 37.9%; *p* = 0.014) were more likely to utilise both teaching approaches than untrained teachers. Within a cross-curriculum approach, FNS was most commonly integrated into HPE (*n* = 215), sciences (*n* = 141) and English (*n* = 131).

##### Teaching materials and information sources

Teachers used a variety of FNS teaching materials and commonly sourced information from teacher-specific websites (*n* = 195, 59.3%), curriculum documents (*n* = 164, 49.8%) and YouTube (*n* = 164, 49.5%) (Table 6). Teachers were more likely to create their own resources (*n* = 145, 44.1%) than use information from government health (*n* = 110, 33.4%) or education (*n* = 73, 22.2%) departments or external experts (*n* = 41, 12.5%).

**Table 6:** FNS teaching materials and information sources

	Proportion *n* (%)
**FNS educational resources and materials (** ***n*** ** = 329)**	
Videos	260 (79.0)
Real food/labels	231 (70.2)
Diagrams/posters	218 (66.3)
Websites	211 (64.1)
Powerpoint slides	212 (64.4)
Cooking / food preparation	162 (49.2)
Discussions	157 (47.7)
Books	145 (44.1)
Food label reading / exploration	140 (42.6)
Composting	138 (41.9)
Food models / photos	137 (41.6)
Food tasting	136 (41.3)
Incursions*	93 (28.3)
Garden tours	90 (27.4)
Games	84 (25.5)
Excursions	82 (24.9)
Quizzes	68 (20.7)
Magazines	57 (17.3)
Other	15 (4.6)
**FNS information sources (** ***n*** ** = 329)**	
Websites dedicated to teaching resources	195 (59.3)
Curriculum documents	164 (49.8)
YouTube	163 (49.5)
Other teachers	148 (45.0)
I create my own resources	145 (44.1)
General websites	125 (38.0)
Government health departments	110 (33.4)
Government education departments	73 (22.2)
Social media	46 (14.0)
External experts	41 (12.5)
Other	16 (4.9)

* An incursion is an activity run by an external organisation within the school grounds (for example, guest speaker)

#### Factors influencing FNS education

##### Family engagement

Teachers used a variety of strategies to engage families in FNS activities including via newsletters (50.1%; 95%CI = 45.3%, 54.9%), sending resources home (25.2%; 95%CI = 21.2%, 29.6%), emails (21.3%; 95%CI = 17.6%, 25.5%) and family participation in gardening, composing and cooking activities (19.6%; 95%CI: 16.1%, 23.7%).

##### Barriers

Common barriers to FNS education included inadequate funding (50.1%; 95%CI = 45.3%, 54.9%) inadequate resources and materials (45.0%; 95%CI = 40.3%, 49.9%), lack of school management support (30.0%; 95%CI = 25.8%, 34.6%) or perceptions that this was not the teacher’s role (22.3%; 95%CI = 18.5%, 26.6%). Of 85 respondents who described additional barriers to FNS education, 68% (*n* = 58) spontaneously reported lack of time/curriculum overcrowding. Only 11.1% (95%CI = 8.4%, 14.6%) of teachers did not experience barriers to teaching FNS.

##### Funding

Less than a third of teachers had access to funding for FNS class activities (29.8%; 95%CI = 25.6%, 34.4%) and fewer had funding for training (19.9%; 95%CI = 16.3%, 24.0%). Teachers with access to funding for FNS activities and training were more likely to be from non-government schools (*p* = 0.008, *p* = 0.034 respectively) and schools with curriculum policies about FNS education (41.5% vs. 18.7%; *p* < 0.001 and 34.8% vs. 11.9%; *p* < 0.001 respectively). There was no association between access to funding and school size, rurality or SEP. Most teachers reported they would participate in training about FNS education if it were paid for (88.9%; 95%CI = 85.4%, 91.6%).

##### Policies

Only 28.6% of teachers (95%CI = 24.4%, 33.1%) reported their school had policies about teaching FNS in the curriculum. Non-government schools were more likely to have such policies in place than government schools (*p* = 0.047).

## Qualitative results

Five themes were constructed from thematic analysis of open-ended questions. These themes extend on the quantitative results reported above and focus on respondent perceptions about the importance of FNS education and family, curriculum, teacher and school environment factors that influence FNS education practices.

### Importance of FNS education

Teachers discussed nuanced perceptions about the importance of FNS education. Nutrition education was considered important *to promote good health and to assist in prevention of future lifestyle diseases*_[ID−117]_. Some teachers described *the link to brain health and learning*_[ID−297]_, which *helps with emotional regulation*_[ID−239]_, and *makes a better learner*,* less sick days*_[ID−209]_. Food education was considered a skill building tool. One teacher described this as *being able to develop skills in preparing and cook*[ing] *foods*,* planning and budgeting meals… Being able to navigate through critical and creative thinking messages in the food environment*_[ID−254]_.

Teachers who discussed food sustainability emphasised social responsibility:

I think sustainability is so important to teach our children ‘waste not, want not’. I am very worried about the generation of children that don’t know where their food has come from, or how to cook it in a healthy way to fuel their bodies._[ID−118]_

### Family factors

Several teachers described FNS education as a parenting responsibility, expressing frustration about expectations on teachers to ‘parent’ students.

As a society we need to stop this thinking that school is the catch all solution for every social problem or issue. Parents [are] the first educators of their children and the ones who decide what they eat - as they should be. It is not the place of a teacher to be providing the education let alone policing this_[ID-124]_.

Food and nutrition… extends beyond teaching and into parenting / social work… For students who have food insecurity, it has to be a social welfare case NOT an education / teacher role_[ID−6]_.

Some considered family food practices to undermine school-based FNS education. However, they noted complex societal factors that influence family food behaviours and needing to respect family food values.

In rural communities with low SES [socioeconomic status] it is difficult to change values and thinking about food and nutrition. Generally parents don’t want to change practices as they don’t see the value in eating good food, and don’t want to take time to prep it. Using pre-packed foods is too easy an option. They are swayed by novelty rather than value and impact of food on brain and body_[ID−298]_.

Even though the parents support the teaching of healthy eating, they are often time poor and take the path of what is easiest for them. Sometimes it is also down to the expense of providing healthy choices all the time_[ID−284]_.

I also truly believe that different families have different ideals related to food and I don’t… have [the] right to tell their children that their lunch boxes are unhealthy._[ID−39]_

For other teachers it was important to engage families in their school’s FNS activities, particularly through food-based education:

Teaching families. It’s not valuable for me to spend hours teaching 6 year olds this when it’s their parents who are filling their lunchboxes. That’s where the focus needs to be - partnership with kids and parents. If it’s just kids, its taking my precious teaching time for very little return on investment_[ID−406]_.

Parents are the gate keepers when it comes to purchasing food, so an initiative that involves everyone would be good_[ID−24]_.

### Curriculum factors

The curriculum was an important focus area, as one teacher explained:

The syllabus is our core document. Teaching outside the scope of those documents is extra workload that is not required in primary school_[ID−198]_.

However, the curriculum was perceived to be overcrowded with the breadth of government-mandated subjects and prioritisation of literacy and numeracy leaving little room for other learning areas:

Too much focus on higher literacy and numeracy and not enough on life skills, well being, and climate change / sustainability. We are just told to focus on the core subjects as a priority_[ID−48]_.

Teachers expressed inconsistent views about the presence of FNS education in government curricula. For example, one explained *Food and nutrition education is an integral part of PDHPE* [Personal Development, Health and Physical Education] *curriculum in every stage of primary*,* and in some science units*_[ID−86]_ whilst another stated it *is not a subject that is actually included in the teaching curriculum*,* which is disappointing*_[ID−183]_.

Nonetheless, many teachers agreed FNS should be in the curriculum, and described the need for *more government funding and a higher focus in the curriculum*_[ID−345]_ with *clearly stated objectives and assessable elements that are reported on*_[ID−137]_ and schools being *mandated to teach X hours per week*_[ID−72]_.

Whilst some teachers emphasised a less crowded curriculum that prioritises FNS education would enable them to integrate this within their teaching, others used FNS education to address core curriculum components:

I teach a patch to plate program where we have embedded science, maths and literacy into our gardening and cooking program…we have shown that through garden and cooking you can cover a lot of Australian curriculum areas including technology, art, science, math, English, cross curriculum priorities, HASS etc_[ID−175]_.

Teachers frequently explained that having easy access to shelf-ready, curriculum-aligned scope and sequence resources would enable FNS education in the classroom.

### Teacher factors

Teachers described feeling overworked and stressed, as one experienced teacher explained:

Teachers are already swamped by the huge amount of content in KLAs [Key Learning Areas] without adding another life skill for teachers to implement. As a teacher of 34 years, I have seen an increase in workload with things like breakfast club and gardening being added. Just because something is worthwhile, important or being neglected by parents does not mean teachers should have it added to their already onerous load_[ID−149]_.

Some considered FNS education outside their scope of practice:

It’s not my job. I’m a teacher and I refuse to take on anymore. There is barely enough time to cover the KLAs. I am not a placebo parent or social worker_[ID−145]_.

However, others were interested in promoting healthy eating and were involved in FNS activities at school:

I have some obviously unhealthy students that don’t bring healthy lunch and are overweight… this makes them unhappy… I’d love to be part of the solution_[ID−178]_.

My class only receives food and nutrition education at our school as I run the Enviro Club at the school and do it in lunchtimes and with my class_[ID−257]_.

Such activities were often conducted in addition to teachers’ usual workload and at their own financial expense, and were often based on their personal knowledge:

Often programs etc. associated with other areas such as cooking, gardens are run by teachers in their own time at lunch times with teacher[s] paying for any resources themselves_[ID−72]_.

My knowledge about nutrition and food cooking skills comes from my own personal life rather than any formal education_[ID−412]_.

### School environment factors

Time, facilities, funding and training opportunities were important factors discussed by several teachers:

Time it all comes down to time and how to fit it in the curriculum. The government demands we put certain hours into subjects so Maths & English is like 6 h a week where as HPE is 30 min. Thats why food and nutrition get left behind_[ID−222]_.

If schools had endless money and resources we all would have sustainable food producing gardens and staff to teach this. It is all about inadequate funding_[ID−22]_.

Just upskilling staff in nutrition but then also giving us the resources to teach in an engaging way would be so impactful on the students_[ID−308]_.

School values and learning priorities were also common themes, as one teacher explained:

Our school is strongly driven by maintaining and improving results in literacy and numeracy. Anything other than that is viewed as a very very very distant second place_[ID−72]_.

Teachers described several factors that would enable FNS education at school including *extra funding*,* change in perspectives around what is valuable for student to learn*_[ID−13]_, *allocated time in the timetable*_[ID−372]_, and *professional learning… that highlight*[s] *the pedagogy of teaching food skills for both graduate and experienced teachers.* Teachers acknowledged this would need *support from the education department and support from school management*_[ID−97]_.

## Discussion

This study analysed current approaches to FNS education in Australian primary schools. The study was novel in that it investigated current practices outside the intervention setting with an integrated focus food sustainability. Thus, we aimed to fill a gap in current literature which largely focuses on school-based nutrition interventions.

Teachers in our study considered FNS education to be important. To our knowledge, this is the first study where teachers have reported FNS education is equally important to almost all mandatory curriculum subjects. Despite this, FNS was generally taught infrequently (1–2 times per term or 1–2 times per year), with teachers reporting that not enough time is spent on FNS education. This is similar to overseas reports where time spent on FNS education has ranged from < 1–13 h per school year [36, 57, 58]. Consistent with previous literature, teachers discussed having little time to teach FNS [37, 41, 42, 59–61] particularly due to competing priorities in an overcrowded mandatory curriculum [60–62]. Teachers discussed feeling stressed due to their high teaching load, which has also been previously reported [63]. Collectively, these findings suggest there is a need for strategies to integrate FNS education into the curriculum without adding to teacher overload.

In our study, several teachers suggested that food education be mandated within the curriculum. Whilst the Australian Curriculum includes food-related content through the learning areas health and physical education, sciences and technologies, it is not mandatory [33]. Whilst advocacy to integrate FNS education into curricula has emerged internationally [23–25], perceptions that the Australian curriculum is already overcrowded suggests that mandating food-related content may increase teacher workload and stress, or result in deprioritisation of other curriculum subjects.

A mechanism to address this is to integrate FNS education into mandatory curriculum subjects using a cross-curriculum approach. Indeed evidence suggests building nutrition education into mathematics, science, literacy and social studies improves student nutrition and academic outcomes [64, 65] and saves teachers’ time [65]. In the Australian curriculum, sustainability is embedded as a cross-curriculum theme [66], presenting a leverage point for integrating food sustainability specifically. However, this is unlikely to extend to broader food and nutrition education. Moreover, consolidating FNS education within the mandatory curriculum would likely require school and government leadership support, shifts in curriculum policies and dedicated funding for resources and training. Despite these hurdles, refocusing mandatory curricula to integrate FNS education is an important next step for public health research, policy and practice.

In our study most teachers agreed they should role model and encourage healthy eating for students, though almost a quarter reported it is not their role to teach FNS at school. Several teachers commented this was a parenting responsibility. This dissonance reflects discordance in published literature where teachers have perceived it to be either their role [42, 60] or the role of parents [67, 68] to teach students about food and nutrition. Moreover, parents have reported nutrition education to be part of a teacher’s responsibility [67]. Collectively, this suggests both need and opportunity to balance these perceptions, including converging opposing mental models about the division of responsibility for FNS education between families and teachers.

Teachers in our study reported high levels of understanding, confidence, knowledge and skills to teach FNS, though few had undertaken training in this field. Despite this, teachers relied on their personal and professional judgement when considering credibility of teaching materials and were more likely to develop their own resources than to use information from government departments or external experts. This raises concern about the reliability of FNS education provided to students, particularly given the large amount of misinformation in the public domain about food, nutrition and what constitutes sustainable food production [69].

Intervention studies have shown that teacher training in food education can improve teachers’ nutrition knowledge [70–72], which is important for ensuring quality and accuracy of the education provided to students. A novel finding in our study was that teachers who had undertaken training were more likely to teach FNS more frequently and to use both stand-alone and cross-curriculum approaches. This may indicate that teachers trained in FNS education have more skill and capacity to teach this using a variety of strategies and approaches. These findings suggest that training teachers to teach FNS is likely to be an effective strategy for supporting credible and appropriate FNS education in the classroom. More research is needed to determine the type of training, including concepts and delivery methods that would adequately qualify teachers to teach FNS education. Notably, this should encompass the multiple complex aspects of food systems (for example, environmental, economic and social) beyond traditional evidence-based information about diet, nutrition and health.

Inadequate funding was an identified barrier to FNS education in our study, as has been observed in previous literature [37]. However, we found a significant association between access to funding and both teacher training and frequency of FNS education, indicating funding could facilitate FNS education in the primary school setting. Whilst less than a third of teachers had access to funding for FNS training or activities, those that did have funding were more likely to be from schools with policies about teaching FNS in the curriculum. The presence of FNS-related policies was also a predictor for increased frequency of FNS education and teacher training in both nutrition and food sustainability education. This aligns with existing literature which suggests school based food literacy initiatives are more likely to be effective when they are supported by policy measures [16]. This may be because strong policy for FNS education within the school setting is likely to help consolidate and normalise FNS education in everyday school life, and ensure its ongoing presence regardless of staff turnover and differing perspectives about its role. Despite the importance of policies to support FNS education at school, less than a third of teachers in our study reported their school had such policies in place.

In our study, teachers from non-government schools (that is, independent or Catholic schools) were more likely to report several attributes that facilitated FNS education. These teachers were more likely to be trained to teach food sustainability and to be from schools with policies in place about teaching FNS in the curriculum. Whilst there is no known reason for this, we posit this may be because in Australia, all independent schools and many Catholic schools use the Australian Curriculum (as opposed to state-specific variations) where sustainability is a mandatory cross-curricular theme. Teachers in non-government schools were also more likely to have access to funding for FNS training and activities. This could be due to the wide income disparity observed between public and private schools in Australia. For example, *The State of School Funding in Australia 2017* report states that the total income per student of independent and Catholic schools is significantly higher than in public schools, due to both private fees and increases in government funding within the private school sector [73]. This may mean that non-government schools have more disposable income to spend on ‘extra’ activities including those related to FNS education.

### Strengths and limitations

Our study included a novel focus on food sustainability education, and we drew on published literature to explain our interpretation of this concept. However, there is no agreed definition of food sustainability education in the literature, which may leave this term open for interpretation by respondents. Establishing a more comprehensive definition of food sustainability education that includes elements beyond environmental sustainability (for example, financial and social factors) is an important area for future research. Whilst the survey tool utilized was based on published literature and tested for face validity, we did not explicitly test for construct or content validity or reliability.

Our study had a large sample size, indicating that results are likely to echo broader perspectives of primary school teachers. Most participants were female and teaching in government schools, reflecting Australia’s primary school teaching workforce within which 82% of teachers are female [74] and 70% of schools are government schools [75]. Most participants were living and teaching in high socioeconomic position areas in Victoria which may impact generalisability of our findings to the broader population of Australian teachers. However, the sample size was large enough to allow for estimates of proportions with high precision. The voluntary nature of the study and moderate completion rate could have resulted in self-selection bias where only particularly interested participants may have completed the survey. However, the large sample size meant that participating teachers had a breadth of teaching experience and taught across all primary school year levels and at schools of varying sizes, indicating the perceptions of a diverse group of Australian teachers were reflected in the survey findings.

### Implications for research and practice

Findings from this study have several implications for research and practice. Teacher reports of the importance of FNS education and requirements for funding, training, resources and leadership support offer a legitimate basis for advocacy according to the expressed needs of teachers themselves. Public health advocates, education departments and policy makers can leverage these findings to promote the integration of FNS education within mandatory federal and state curricula and strengthen the position of FNS education within the primary school education system. Future research should explore best-practice strategies for supporting the integration of FNS education in primary schools, including through teacher support, training, funding, resources and curriculum alignment, with a clear line of sight from research to practical implementation.

## Conclusion

This study provides insights from primary school teachers about their perceptions and practices regarding FNS education, as well as factors influencing FNS education in the primary school setting. We found that teachers consider FNS education to be as important as most mandatory curriculum subjects, but their capacity to teach this is limited by a variety of factors including time, funding, resources and training as well as perceptions about overcrowding within the curriculum. Strengthening FNS education in the Australian primary school sector is an important next step for public health research, policy and practice, particularly the integration of FNS education within the curriculum and support mechanisms for teachers to implement this in practice.

## Electronic supplementary material

Below is the link to the electronic supplementary material.

Supplementary Material 1

Supplementary Material 2

## Data Availability

The datasets used and/or analysed during the current study are available from the corresponding author on reasonable request.

## Abbreviations

CI: Confidence interval
FNS: Food, Nutrition and Sustainability
HASS: Humanities and Social Sciences
HPE: Health and Physical Education
IRSAD: Index of Relative Socioeconomic Advantage and Disadvantage
KLA: Key Learning Area
PDHPE: Personal Development, Health and Physical Education
REDCap: Research Electronic Data Capture
SEP: Socioeconomic Position
STROBE: Strengthening the Reporting of Observational Studies in Epidemiology
