# Correction: Food, nutrition and sustainability education in Australian primary schools: a cross-sectional analysis of teacher perspectives and practices

**DOI:** 10.1186/s13690-024-01475-2

**Published:** 2024-12-16

**Authors:** Jessica V. Kempler, Claire Margerison, Janandani Nanayakkara, Alison Booth

**Affiliations:** Deakin University Institute for Physical Activity and Nutrition, 75 Pigdons Road, Waurn Ponds, VIC 3216 Australia

Correction: *Arch Public Health***82**, 222 (2024).

10.1186/s13690-024-01449-4

Following publication of the original article [1], the authors requested to modify the order of the authors from Jessica V. Kempler, Alison Booth, Claire Margerison and Janandani Nanayakkara to Jessica V. Kempler, Claire Margerison, Janandani Nanayakkara and Alison Booth.

The original article [1] has been updated.
