# Supplementary material for: Food, nutrition and sustainability education in Australian primary schools: a cross-sectional analysis of teacher perspectives and practices
Source: Arch Public Health. 2024 Nov 22;82:222. doi: 10.1186/s13690-024-01449-4 (PMC11583557; doi:10.1186/s13690-024-01449-4)
Supplement: Supplementary file 1 — Supplementary Material 1 [file 13690_2024_1449_MOESM1_ESM.docx]

**Supplement to *Food, nutrition and sustainability education in Australian primary schools: A cross-sectional analysis of teacher perspectives and practices***

Survey questions and response

**Screening question:** Are you currently a classroom teacher of primary level students (Foundation - Grade 6) at a school in Australia?

- Yes
- No

Definitions

In this survey, food and nutrition education includes:

- *Nutrition education* which means teaching students about nutritious foods and eating patterns that promote good health and reduce the risk of disease.
- *Food skills education* which means teaching students to plan, purchase, store, prepare and cook nutritious food.
- *Food sustainability education* which means teaching students to use nutritious food in a way that does not waste natural resources and can be continued into the future without harming our environment or health.

Throughout this survey, the term 'food' includes food and drinks.

**Domain 1: Teacher perceptions and attributes regarding FNS education**

These statements are about the role of primary schools and primary school teachers in food and nutrition education. Please indicate how much you agree with each statement.

|  | Strongly agree | Agree | Neither agree nor disagree | Disagree | Strongly disagree |
| --- | --- | --- | --- | --- | --- |
| Primary schools should: | | | | | |
| Teach food and nutrition as part  of the curriculum | ○ | ○ | ○ | ○ | ○ |
| Have policies in place about food  and nutrition | ○ | ○ | ○ | ○ | ○ |
| Primary school teachers should: | | | | | |
| Role model healthy eating to  students | ○ | ○ | ○ | ○ | ○ |
| Encourage students to eat  healthy food | ○ | ○ | ○ | ○ | ○ |

How important is it to teach primary school students about:

|  | Extremely important | Very important | Moderately important | Slightly important | Not at all important |
| --- | --- | --- | --- | --- | --- |
| Nutrition | ○ | ○ | ○ | ○ | ○ |
| Food skills | ○ | ○ | ○ | ○ | ○ |
| Food sustainability | ○ | ○ | ○ | ○ | ○ |

How important is food and nutrition education for primary school students compared to these other subjects / learning areas:

|  | Food / nutrition is more important than this subject | Food / nutrition is as  important as this subject | Food / nutrition is less important than this subject |
| --- | --- | --- | --- |
| English (including language,  literature and literacy) | ○ | ○ | ○ |
| Mathematics | ○ | ○ | ○ |
| Science | ○ | ○ | ○ |
| Health and physical education | ○ | ○ | ○ |
| Humanities and social sciences | ○ | ○ | ○ |
| The arts (including dance, drama, art, music) | ○ | ○ | ○ |
| Technologies | ○ | ○ | ○ |
| Languages | ○ | ○ | ○ |
| Mental health | ○ | ○ | ○ |

These statements are about your confidence, knowledge and enjoyment about teaching food and nutrition to primary school students. Please indicate how much you agree with each statement.

|  | Strongly agree | Agree | Neither agree nor disagree | Disagree | Strongly disagree |
| --- | --- | --- | --- | --- | --- |
| I have a good understanding of: | | | | | |
| Nutrition | ○ | ○ | ○ | ○ | ○ |
| Food skills | ○ | ○ | ○ | ○ | ○ |
| Food sustainability | ○ | ○ | ○ | ○ | ○ |
| I am confident in my ability to teach my students about: | | | | | |
| Nutrition | ○ | ○ | ○ | ○ | ○ |
| Food skills | ○ | ○ | ○ | ○ | ○ |
| Food sustainability | ○ | ○ | ○ | ○ | ○ |
| I have undertaken training and / or professional development to teach my students about: | | | | | |
| Nutrition | ○ | ○ | ○ | ○ | ○ |
| Food skills | ○ | ○ | ○ | ○ | ○ |
| Food sustainability | ○ | ○ | ○ | ○ | ○ |
| I have the knowledge and skills required to teach my students about: | | | | | |
| Nutrition | ○ | ○ | ○ | ○ | ○ |
| Food skills | ○ | ○ | ○ | ○ | ○ |
| Food sustainability | ○ | ○ | ○ | ○ | ○ |
| I enjoy / would enjoy teaching my students about: | | | | | |
| Nutrition | ○ | ○ | ○ | ○ | ○ |
| Food skills | ○ | ○ | ○ | ○ | ○ |
| Food sustainability | ○ | ○ | ○ | ○ | ○ |

Is there anything else that is important for us to know about your / your school's role in food and nutrition education?

**Domain 2: FNS teaching practices**

How frequently are students in your class taught about food and nutrition? This includes as a stand-alone subject or included in other learning areas, and taught by you and / or by others

- A few times a week
- Once a week
- Once a fortnight
- Once or twice a term
- Once or twice a year
- Never but I am interested in this being taught (by me and / or others) to my students
- Never and I am not interested in this being taught (by me and / or others) to my students
- Other (Please specify how frequently food and nutrition is taught to students in your class)

Do you think that enough time is spent (either by you or others) teaching your students about food and nutrition?

- Yes
- No

Who usually teaches food and nutrition to students in your class (including yourself and / or others)?

- Only me
- Me and others (Please specify who teaches food and nutrition to your students, aside from you)
- Only others (Please specify who teaches food and nutrition to your students)

Do you teach food and nutrition education as:

- Stand-alone subjects only
- Both stand-alone and cross-curriculum subjects
- Cross-curriculum subjects only

*Note: 'cross-curriculum' means including food and nutrition education in other subjects / learning areas*

Using a cross-curriculum approach, in which subjects / learning areas do you currently include food and nutrition education? (Tick all that apply)

- English (including language, literature and literacy)
- Mathematics
- Sciences
- Health and physical education
- Humanities and social sciences
- The arts (including dance, drama, arts, music)
- Technologies
- Languages
- Other (Please specify)

*Note: 'cross-curriculum' means including food and nutrition education in other subjects / learning areas*

What sort of resources, materials and activities do you use to teach food and nutrition? (Tick all that apply)

- Videos
- Diagrams / posters
- PowerPoint slides
- Books
- Magazines
- Websites
- Games
- Real food / food labels
- Food models or photos
- Excursions
- Incursions
- Discussions
- Quizzes
- Food tasting
- Food label reading / exploration
- Cooking and / or food preparation
- Garden tours
- Composting
- Other (Please specify)

Where do you obtain educational resources (e.g., activity sheets, lesson plans, project ideas) for teaching food and nutrition? (Tick all that apply)

- Curriculum documents (Please specify)
- Other teachers
- YouTube
- Social media (Please specify)
- Websites dedicated to teaching resources (e.g., Twinkl, TeachStarter, Refresh ED) (Please specify)
- General websites (Please specify)
- External experts (Please specify)
- Government education department (e.g., FUSE website) (Please specify)
- Government health department (e.g., Australian Dietary Guidelines) (Please specify)
- I create my own resources
- Other (Please specify)

How do you make decisions about the credibility of the resources you use to teach food and nutrition?

**Domain 3: Factors influencing FNS education**

Which strategies are used to engage families in your school's food and nutrition activities? (Tick all that apply)

- Newsletters
- Emails
- App messages
- Sending resources home (e.g., recipes)
- Family participation in classroom activities
- Family participation in gardening, composting and cooking activities
- Meetings and information sessions
- Other (Please specify)
- None, and I don't think my school needs to do this (Please provide some examples of what your school could do to engage families in food and nutrition activities)
- None, but I think my school should do this

Which of the following challenges (if any) do you experience about teaching food and nutrition? (Tick all that apply)

- Students are not interested in learning this
- I do not have adequate teaching and learning resources and materials
- I do not have adequate funding
- I do not get support from my school management to teach this
- Parents do not support this being taught at school
- It is not my role to teach this at school
- Other challenges (Please specify)
- I don't experience any challenges about teaching food and nutrition

What would support you to overcome these challenges?

Do you have access to funding for:

|  | Yes | No |
| --- | --- | --- |
| Food and nutrition class activities (e.g., excursions, cooking ingredients, classroom  practical experiences, etc) | ○ | ○ |
| Training and / or professional development about teaching food and nutrition to students | ○ | ○ |

If it were paid for, would you participate in training or professional development about food and nutrition education?

- Yes
- No

Does your school have written policies about teaching food and nutrition in the curriculum?

- Yes
- Unsure
- No

Is there anything else you think is important for us to know about your classroom's practices for teaching food and nutrition to primary school students?

**Demographic questions:**

Please provide the following information about the school you work at:

- State / Territory:
- Postcode:
- Type of school:

Approximately how many primary level students are enrolled at the school you work at?

- Less than 24
- 25-49
- 50-99
- 100-199
- 200-299
- 300-399
- 400-499
- 500-599
- 600-699
- 700-799
- 800-899
- 900-999
- More than 1000

Which grade(s) do you currently teach? (Tick all that apply)

- Foundation / Prep
- Grade 1
- Grade 2
- Grade 3
- Grade 4
- Grade 5
- Grade 6

How long (in years) have you worked as a primary school teacher?

- Less than 1 year
- 6-10 years
- 11-15 years
- 16-20 years
- 21-25 years
- More than 25 years

How long (in years) have you worked in your current teaching role?

- Less than 1 year
- 1-5 years
- 6-10 years
- 11-15 years
- 16-20 years
- 21-25 years
- More than 25 years

What is your residential (home) postcode?

What is your age?

- 18-24 years
- 25-34 years
- 35-44 years
- 45-54 years
- 55-64 years
- 65+ years

How do you describe your gender identity?

- Woman
- Man
- Gender diverse
- Non-binary
- Prefer to self describe
- Prefer not to specify
