# Supplementary material for: Food, nutrition and sustainability education in Australian primary schools: a cross-sectional analysis of teacher perspectives and practices
Source: Arch Public Health. 2024 Nov 22;82:222. doi: 10.1186/s13690-024-01449-4 (PMC11583557; doi:10.1186/s13690-024-01449-4)
Supplement: Supplementary file 2 — Supplementary Material 2 [file 13690_2024_1449_MOESM2_ESM.docx]

**Supplement 2 to *Food, nutrition and sustainability education in Australian primary schools: A cross-sectional analysis of teacher perspectives and practices***

Qualitative analysis codebook

| **Themes** | **Sub-themes** |  |  |  | **References** | |
| --- | --- | --- | --- | --- | --- | --- |
| **1. Importance of FNS education** |  |  |  |  | | **290** |
|  | *Food relationships* | | | | | 74 |
|  |  | Autonomy |  |  | | 16 |
|  |  | Shame |  |  | | 45 |
|  |  | Family circumstances |  |  | | 31 |
|  |  | Not fed at home |  |  | | 3 |
|  |  | Socioeconomic position |  |  | | 27 |
|  | *Food, health, and learning* | | | | | 149 |
|  |  | Cognition |  |  | | 12 |
|  |  | Foundation |  |  | | 14 |
|  |  | Growth |  |  | | 4 |
|  |  | Health |  |  | | 119 |
|  |  |  | Disease |  | | 8 |
|  |  |  | Mental health |  | | 12 |
|  |  |  | Prevention |  | | 36 |
|  |  |  | Unhealthy food |  | | 9 |
|  | *Skills development* | | | | | 40 |
|  |  | Broader influence |  |  | | 11 |
|  |  | Budgeting |  |  | | 3 |
|  |  | Cooking |  |  | | 3 |
|  |  | Critical thinking |  |  | | 1 |
|  |  | Food preparation |  |  | | 11 |
|  |  | Life skills |  |  | | 11 |
|  | *Sustainability* | | | | | 27 |
|  |  | Curriculum priority |  |  | | 7 |
|  |  | Environment |  |  | | 12 |
|  |  |  | Food waste |  | | 2 |
|  |  | Food supply |  |  | | 4 |
|  |  | Scare tactic |  |  | | 1 |
|  |  | Social responsibility |  |  | | 3 |
| **2. Family factors** | | | | | | **259** |
|  | *Child factors* | | | | | 25 |
|  |  | Age appropriate |  |  | | 10 |
|  |  | Broader family influence |  |  | | 6 |
|  |  | Child interest |  |  | | 3 |
|  |  | Child involvement |  |  | | 6 |
|  | *Family as barrier* | | | | | 54 |
|  |  | Lunchbox |  |  | | 17 |
|  |  | Not taught at home |  |  | | 11 |
|  | *Family as enabler* | | | | | 29 |
|  | *FNS is parenting* | | | | | 50 |
|  | *Future family engagement* | | | | | 93 |
|  |  | Canteen |  |  | | 1 |
|  |  | Cooking |  |  | | 15 |
|  |  | Education |  |  | | 5 |
|  |  | Events |  |  | | 13 |
|  |  | Food from home |  |  | | 1 |
|  |  | Food sales |  |  | | 2 |
|  |  | Garden |  |  | | 16 |
|  |  | Homework |  |  | | 2 |
|  |  | Lunchbox reward |  |  | | 1 |
|  |  | Newsletter |  |  | | 19 |
|  |  | Online classes |  |  | | 1 |
|  |  | Recipes |  |  | | 10 |
|  |  | Recycling |  |  | | 1 |
|  |  | Social media |  |  | | 3 |
|  |  | Website |  |  | | 1 |
|  | *Multiculturalism* | | | | | 8 |
| **3. Curriculum factors** | | | | | | **238** |
|  | *Assessment* | | | | | 3 |
|  | *Change curriculum* | | | | | 17 |
|  | *Core priorities* | | | | | 17 |
|  | *Cross curriculum* | | | | | 36 |
|  | *Curriculum resources* | | | | | 72 |
|  | *Government* | | | | | 21 |
|  | *Overcrowded* | | | | | 64 |
|  | *Scope and sequence* | | | | | 3 |
|  | *Specialist subject* | | | | | 5 |
| **4. Teacher factors** | | | | | | **675** |
|  | *Current practices* | | | | | 616 |
|  |  | Activities |  |  | | 51 |
|  |  |  | Discussions |  | | 1 |
|  |  |  | Fruit or veg snack |  | | 7 |
|  |  |  | Health subject |  | | 1 |
|  |  |  | Integrated approach |  | | 2 |
|  |  |  | Kitchen garden compost |  | | 18 |
|  |  |  | Lunch club |  | | 1 |
|  |  |  | Market |  | | 1 |
|  |  |  | Misc |  | | 5 |
|  |  |  | Not or rarely taught |  | | 4 |
|  |  |  | OzHarvest |  | | 1 |
|  |  |  | Routine |  | | 2 |
|  |  |  | Theory v practice |  | | 2 |
|  |  |  | Waste free |  | | 6 |
|  |  | Credibility |  |  | | 257 |
|  |  |  | Align to curriculum |  | | 13 |
|  |  |  | Currency |  | | 4 |
|  |  |  | Discuss with team |  | | 12 |
|  |  |  | Don't assess credibility |  | | 5 |
|  |  |  | Partnerships |  | | 1 |
|  |  |  | Producer |  | | 111 |
|  |  |  |  | Community orgs | | 5 |
|  |  |  |  | Farm | | 2 |
|  |  |  |  | Government | | 43 |
|  |  |  |  | Health professional | | 9 |
|  |  |  |  | Health promotion org | | 7 |
|  |  |  |  | Health websites | | 3 |
|  |  |  |  | Industry | | 3 |
|  |  |  |  | Teacher | | 5 |
|  |  |  |  | Trusted | | 19 |
|  |  |  | Recommended |  | | 1 |
|  |  |  | Relevance |  | | 13 |
|  |  |  | Scientific |  | | 16 |
|  |  |  | Self assessment |  | | 80 |
|  |  |  | Student interest |  | | 1 |
|  |  | Information sources |  |  | | 308 |
|  |  |  | Government websites |  | | 88 |
|  |  |  |  | Dietary guidelines | | 16 |
|  |  |  |  | Education department or curriculum | | 70 |
|  |  |  |  | Health department | | 1 |
|  |  |  | Health or environment organisation |  | | 41 |
|  |  |  | Health professional |  | | 5 |
|  |  |  | Industry organisation |  | | 3 |
|  |  |  | Media |  | | 15 |
|  |  |  | Social media |  | | 14 |
|  |  |  | Teacher specific websites |  | | 142 |
|  |  |  |  | Clickview | | 3 |
|  |  |  |  | Continuum | | 1 |
|  |  |  |  | Cool Australia | | 2 |
|  |  |  |  | Inquisitive | | 4 |
|  |  |  |  | K5 | | 1 |
|  |  |  |  | Lets teach primary | | 1 |
|  |  |  |  | Mappen | | 1 |
|  |  |  |  | Scootle | | 1 |
|  |  |  |  | Teach starter | | 35 |
|  |  |  |  | Teachers pay teachers | | 11 |
|  |  |  |  | Teachthis | | 4 |
|  |  |  |  | Top teacher | | 2 |
|  |  |  |  | Topteacher | | 3 |
|  |  |  |  | Twinkl | | 73 |
|  | *Not teacher responsibility* | | | | | 18 |
|  | *Teacher expertise* | | | | | 9 |
|  | *Teacher led activities* | | | | | 12 |
|  | *Teacher part of solution* | | | | | 1 |
|  | *Teacher personal experiences* | | | | | 5 |
|  | *Teacher stress* | | | | | 12 |
|  | *Teacher suitability* | | | | | 2 |
| **5. School factors** | | | | | | **479** |
|  | *Facilities* | | | | | 61 |
|  |  | Canteen |  |  | | 6 |
|  |  | Food access |  |  | | 1 |
|  |  | Lack of facilities |  |  | | 11 |
|  |  | Need for facilities |  |  | | 43 |
|  | *Funding* | | | | | 93 |
|  |  | Funding as barrier |  |  | | 13 |
|  |  | Need for funding |  |  | | 80 |
|  | *Professional development* | | | | | 60 |
|  | *Risk* | | | | | 20 |
|  |  | Allergy |  |  | | 12 |
|  | *School activities to promote FNS education* | | | | | 24 |
|  |  | Breakfast club |  |  | | 2 |
|  |  | Fun |  |  | | 2 |
|  |  | Lesson type and class |  |  | | 6 |
|  |  | Lunch program |  |  | | 4 |
|  |  | Policies |  |  | | 4 |
|  | *School demographic* | | | | | 22 |
|  |  | Community |  |  | | 2 |
|  |  | Remoteness and location |  |  | | 4 |
|  |  | Size |  |  | | 1 |
|  |  | Specialist school |  |  | | 15 |
|  |  |  | Special needs children |  | | 14 |
|  | *School values* | | | | | 48 |
|  |  | Ethos |  |  | | 2 |
|  |  | Leadership support |  |  | | 27 |
|  |  | Priorities |  |  | | 19 |
|  | *Staffing* | | | | | 30 |
|  |  | Adequate staffing |  |  | | 14 |
|  |  | Professional or specialist |  |  | | 16 |
|  | *Student challenges* | | | | | 2 |
|  | *Time* | | | | | 103 |
|  | *Whole school community* | | | | | 16 |
